# Assessment of dimensional stability of novel VPES impression material at different time intervals with standard disinfectants

**DOI:** 10.1186/s12903-024-04323-5

**Published:** 2024-05-18

**Authors:** Bhavna M. Ahuja, Karuna G. Pawashe, Pronob Kumar Sanyal, Mohammed A. Al-Qarni, Nasser M. Alqahtani, Saeed M. Alqahtani, Abdul Razzaq Ahmed, Mohasin Abdul Khader, Asim Elsir Elmahdi, Saurabh Chaturvedi

**Affiliations:** 1 Department of Prosthodontics and Crown & Bridge, Terna Dental College, Nerul, Navi Mumbai, Maharashtra 400706 India; 2 Department of Prosthodontics and Crown & Bridge, School of Dental Sciences, Krishna Vishwa Vidyapeeth, Deemed to be University, Karad, Satara, Maharashtra India; 3 Department of Restorative Dental Sciences College of Dentistry, King Khalid University, P.O.Box 3263, 61471 Abha, Saudi Arabia; 4 Department of Prosthetic Dentistry, College of Dentistry, King Khalid University, Abha, Saudi Arabia; 5 Division of Periodontics, Department of Periodontics and Community Dental Sciences (PCS), College of Dentistry, King Khalid University, Abha, Saudi Arabia; 6 Department of Dental Research Cell, Dr. D. Y. Patil Dental College and Hospital, Dr. D. Y. Patil Vidyapeeth, Sant-Tukaram Nagar, Pimpri, Pune, 411018 India

**Keywords:** Vinyl polyether silicone (VPES), Biomaterials, In-vitro study, Novel materials, Dental materials, Glutaraldehyde, Sodium hypochlorite, Biotechnology

## Abstract

**Background:**

Vinyl polyether silicone (VPES) is a novel impression biomaterial made of a combination of vinyl polysiloxane (VPS) and polyether (PE). Thus, it is significant to assess its properties and behaviour under varied disinfectant test conditions. This study aimed to assess the dimensional stability of novel VPES impression material after immersion in standard disinfectants for different time intervals.

**Methods:**

Elastomeric impression material used –medium body regular set (Monophase) [Exa’lence GC America]. A total of 84 Specimens were fabricated using stainless steel die and ring (ADA specification 19). These samples were distributed into a control group (*n*=12) and a test group (*n*=72). The test group was divided into 3 groups, based on the type of disinfectant used - Group-A- 2% Glutaraldehyde, Group-B- 0. 5% Sodium hypochlorite and Group-C- 2% Chlorhexidine each test group was further divided into 2 subgroups (*n*=12/subgroup) based on time intervals for which each sample was immersed in the disinfectants – subgroup-1- 10 mins and Subgroup 2- 30 mins. After the impression material was set, it was removed from the ring and then it was washed in water for 15 seconds. Control group measurements were made immediately on a stereomicroscope and other samples were immersed in the three disinfection solutions for 10 mins and 30 mins to check the dimensional stability by measuring the distance between the lines generated by the stainless steel die on the samples using a stereomicroscope at x40 magnification.

**Results:**

The distance measured in the control group was 4397.2078 µm and 4396.1571 µm; for the test group Group-A- 2% Glutaraldehyde was 4396.4075 µm and 4394.5992 µm; Group-B- 0. 5% Sodium hypochlorite was 4394.5453 µm and 4389.4711 µm Group-C- 2% Chlorhexidine was 4395.2953 µm and 4387.1703 µm respectively for 10 mins and 30 mins. Percentage dimensional change was in the range of 0.02 – 0.25 for all the groups for 10 mins and 30 mins.

**Conclusions:**

2 % Glutaraldehyde is the most suitable disinfectant for VPES elastomeric impression material in terms of dimensional stability and shows minimum dimensional changes as compared to that of 2% Chlorhexidine and 0.5% Sodium hypochlorite.

## Introduction

The impression procedure is one of the most important steps in dentistry and its accuracy determines the success of the prosthesis. Even though, with the development of digital technologies, the use of digital impressions is growing but still, conventional impression techniques hold a major part in dentistry work, as it is a more user-friendly method of recording tissue details both in dentulous and edentulous subjects [1–7] .

The most important property required in impression material (IM) is accuracy in recording the details of tissues [8, 9]. The surface characteristics of the IM, such as dimensional stability, surface roughness, hydrophilicity, and detailed replication, are responsible for the impression accuracy. Establishing a standard disinfection procedure for a particular *IM* is crucial because, in dentistry, the accuracy of impression and disinfection procedures go hand in hand.

In the current situation where recently humankind has faced COVID-19, alng with other pathogens (e.g., Candida albicans, streptococci, Escherichia coli, Mycobacterium tuberculosis, staphylococci, hepatitis C virus, and Herpes simplex virus,) [7, 9, 10], special care should be paid for the safety as saliva can be an ideal ecosystem for the growth of communicable, infectious, contagious microbes [11]. The dental impression is one of the prime sources of cross-contamination from an infected patient to clinicians and further to laboratories [9, 10]. Thus, it is very important to disinfect the impression in a way that its accuracy is also maintained, and pathogens are also destroyed.

At present there is no documented “gold standard” method for the disinfection of dental impressions. Various methods described are immersing, spraying, ultraviolet C (UVC), and gaseous ozone etc, among the immersion technique is accepted as the most effective to prevent cross-contamination and spraying techniques are more efficient in the clinical setting, in both common chemicals used include 2% Glutaraldehyde, 0.5 % of sodium hypochlorite and 2% Chlorhexidine [10]. However, these chemical disinfectants have an impact on dental impression material qualities, which in turn affects dental cast quality and accuracy as well as finished prosthetic or orthodontic works [12].

Recently a new impression material has been developed with promising results in accuracy and hydrophobicity. Vinyl polyether silicone (VPES), a novel elastomeric impression material, offered a variety of viscosities and setting times. The manufacturer described it as a blend of polyether (PE) and vinyl polysiloxane (VPS) [13]. This substance is a blend of silicon dioxide (30%–65%), vinyl dimethylpolysiloxane (10%–50%), and methyl hydrogen dimethylpolysiloxane (3%–10%). It comes in low-, medium-, and high-body viscosity levels and offers superior wettability along with improved mechanical and flow characteristics. The accuracy of VPES impressions for finer details on moist dental surfaces or in the gingival sulcus has long been known [14].

According to the manufacturer data sheet for VPES, PE makes up 5% to 20% of the material's total composition, which increases the material's hydrophilicity and improves final impressions in situations where humidity is a concern, such as when moisture control is challenging due to excessive salivation or the presence of minute bleeding [13, 15]. The manufacturer of EXA'lence impression material states that even after the impression has been poured for up to two weeks, the PE-containing material exhibits exceptional dimension stability. In previous studies, the EXA'lence 370 monophase regular set was investigated by performing indirect measurements on casts poured from VPES impressions [1] and the heavy body fast set was investigated by making direct measurements on impression discs [1]. Nassar et al [2]found that there was a definite dimensional change in VPES impression discs after disinfection in glutaraldehyde for 30 mins and prolonged storage for 2 weeks complied with ANSI/ADA standards. Shahab Ud Din et al [16] conducted a study to evaluate linear dimensional changes of synthesized Tetra-functional (dimethylsilyl) orthosilicate (TFDMOS) containing

Polyvinylsiloxane (PVS) impressions following sodium hypochlorite disinfection and concluded that experimental PVS had linear dimensional changes within the ISO 4823; 2015 recommended range. However, extended immersion can negatively affect the linear dimensions. Similarly, Noorhayati R. Mohd et al [17] compared the dimensional stability of two elastomeric impression materials, namely polyvinyl siloxane (PVS) and vinyl siloxanether (VSE), subjected to chemical immersion and microwave irradiation for disinfection and found that. VSE exhibited excellent dimensional stability than PVS under both chemical immersion and microwave irradiation. However, there is a lack of literature when it comes to the use of other standard disinfectants especially with VPES. So, the present study was performed with the aim to determine the effect on the dimensional stability of VPES impression after immersing in three disinfectants 2% Glutaraldehyde, 0.5 % sodium hypochlorite and 2% Chlorhexidine. The null hypothesis formulated that there will be no change in the dimensional stability of VPES impression after immersing it in 3 different disinfectants.

## Materials and methods

**Fig. 1:**
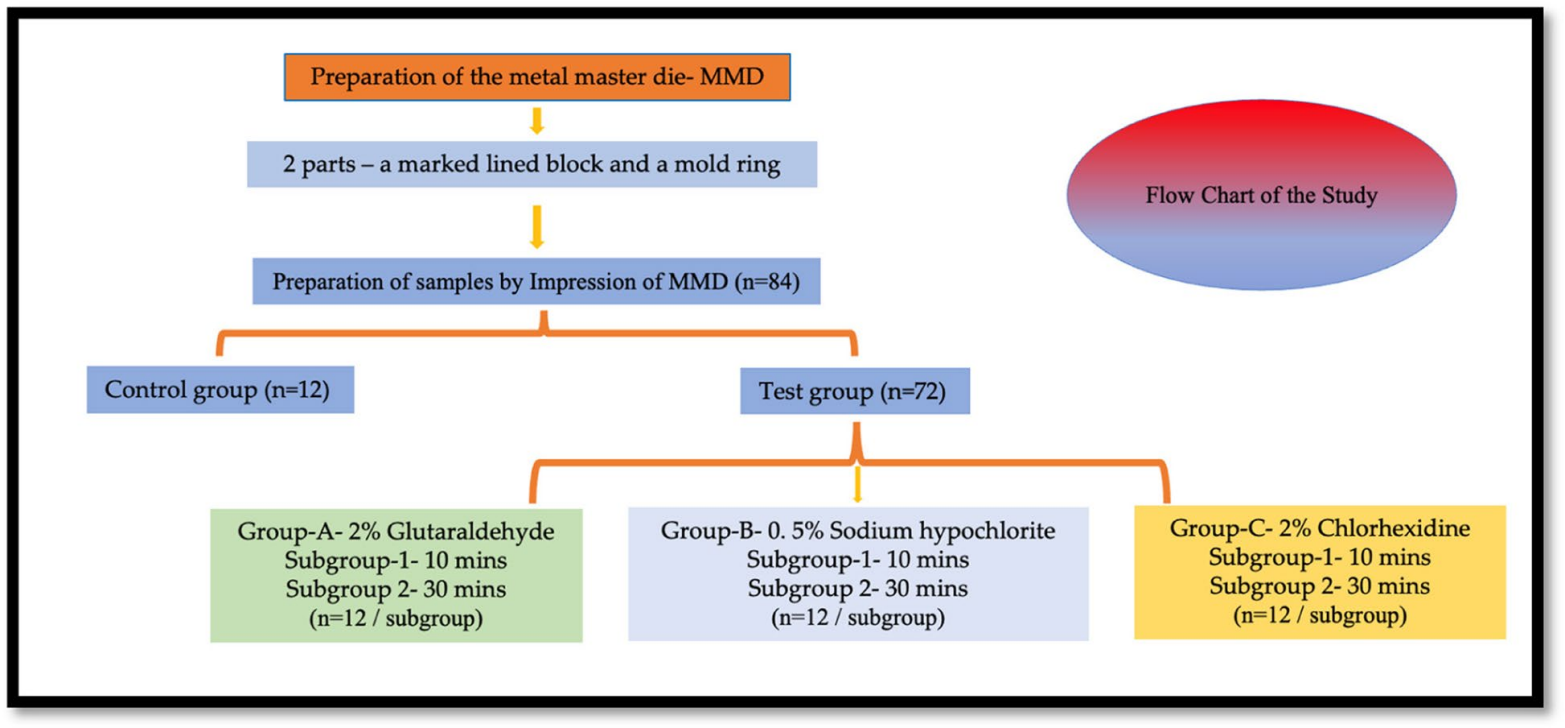
Flow chart of the study

The present cross-sectional study was conducted in the Department of Prosthodontics, School of Dental Sciences, Krishna Institute of Medical Sciences, India. and was approved by the institute’s ethical committee. To standardize the study protocol, a synchronized flowchart (Fig. 1) was prepared for the fabrication of samples in the study. This research was conducted to determine the effect on the dimensional stability of VPES impression after immersing in 3 disinfectants 2% Glutaraldehyde, 0.5 % of sodium hypochlorite and 2% Chlorhexidine. The objective was to decide and recommend a disinfectant which can be used for VPES impression material without affecting its accuracy.

### Preparation of the metal master die

**Fig. 2:**
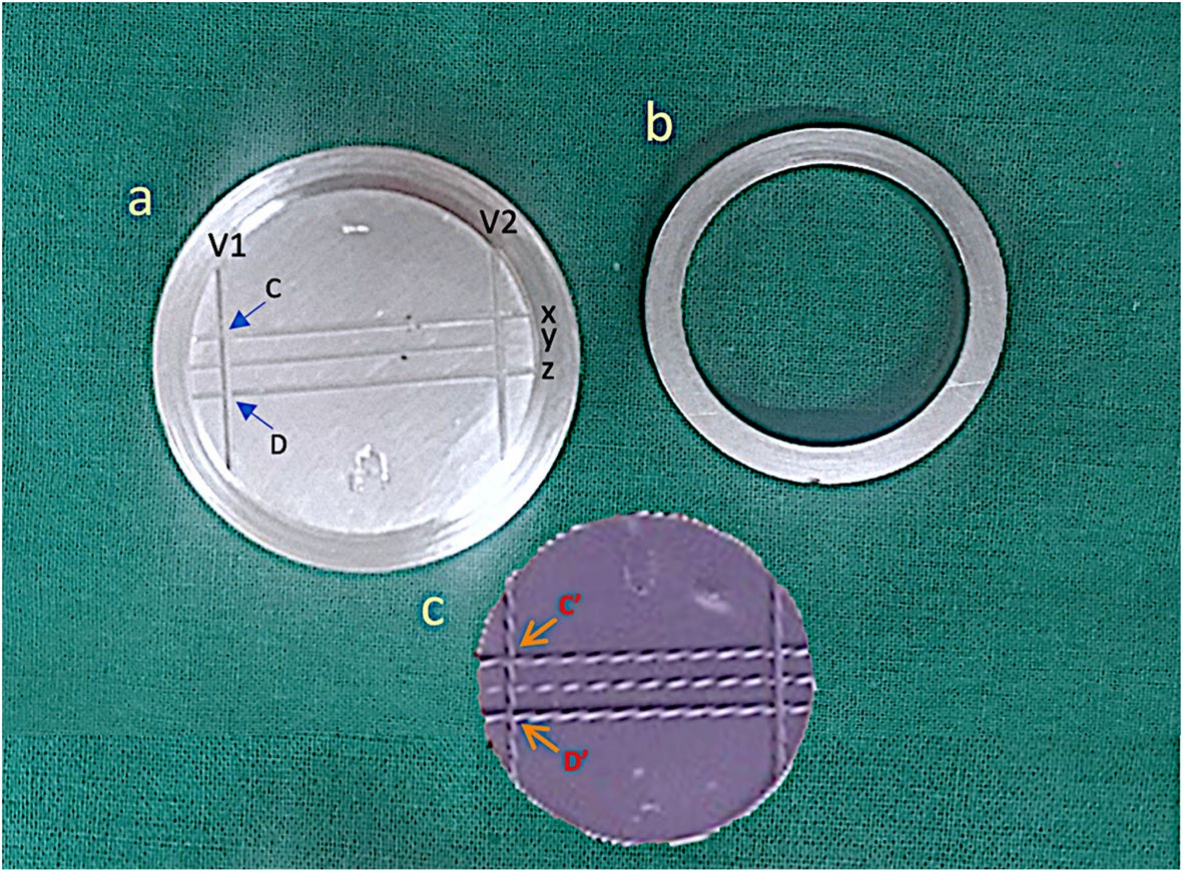
**a-**Stainless steel (SS) metal master die (MMD) showing the horizontal X,Y,Z lines, V1, V2 vertical lines and C , D points; **b** corresponding ring of metal master die (MMD) ; **c** Representative VPES impression disc showing C’ , D’ point corresponding to C and D on metal master die (MMD).

In this study, a stainless steel (SS) metal master die (MMD) was made as per ADA specification number 19 [18] (Fig. 2). The MMD comprised 2 parts – a marked lined block and a mold ring. The dimensions lined block were 31mm in height and 38mm in width. A 3mm height and 29.97mm diameter stage was made on the sides of the MMD and a metal mold ring was fitted to it. The MMD entails 3 parallel horizontal lines engraved on the surface of the MMD viz. X, Y and Z and two vertical lines marked as V1 and V2. The dimensions of the diameter of the mold ring were 38mm (outer ring), 30mm (inner ring) and 6mm height which fits around the borders as a mold for the impression material. The stereomicroscope (Z4 Zoom) (Caliper Pro version - 4.6) with x40 magnification (0.0001 mm submicron precision) was used to determine the distance between the X and Z parallel lines, between 2 predesignated points mentioned to C and D (corresponds to the intersection of V1 vertical line with X and Z horizontal lines. ) which was approximately 5mm [19]. The same points when measured in impression discs samples were designated as C’ and D’ (Fig. 2). This made it possible to conduct the study under identical circumstances, allowing other researchers to compare the results to other materials under the same or a similar set of testing settings.

### Group division and sample size

The sample size calculation was done to obtain a power of 90% with 0.5 alpha error and 0.2 effect size, as per an earlier study [19]. A total of 84 samples of VPES impression material were made in the form of discs. These samples were distributed into a control group (*n*=12) and a test group (*n*=72). The test group was divided into 3 groups, based on the type of disinfectant used - Group-A- 2% Glutaraldehyde, Group-B- 0. 5% Sodium hypochlorite and Group-C- 2% Chlorhexidine each test group was further divided into 2 subgroups (*n*=12/subgroup) based on time intervals for which each sample was immersed in the disinfectants – subgroup-1- 10 mins and Subgroup 2- 30 mins. The samples were prepared from the VPES impression material.

### Preparation of the samples

**Fig. 3:**
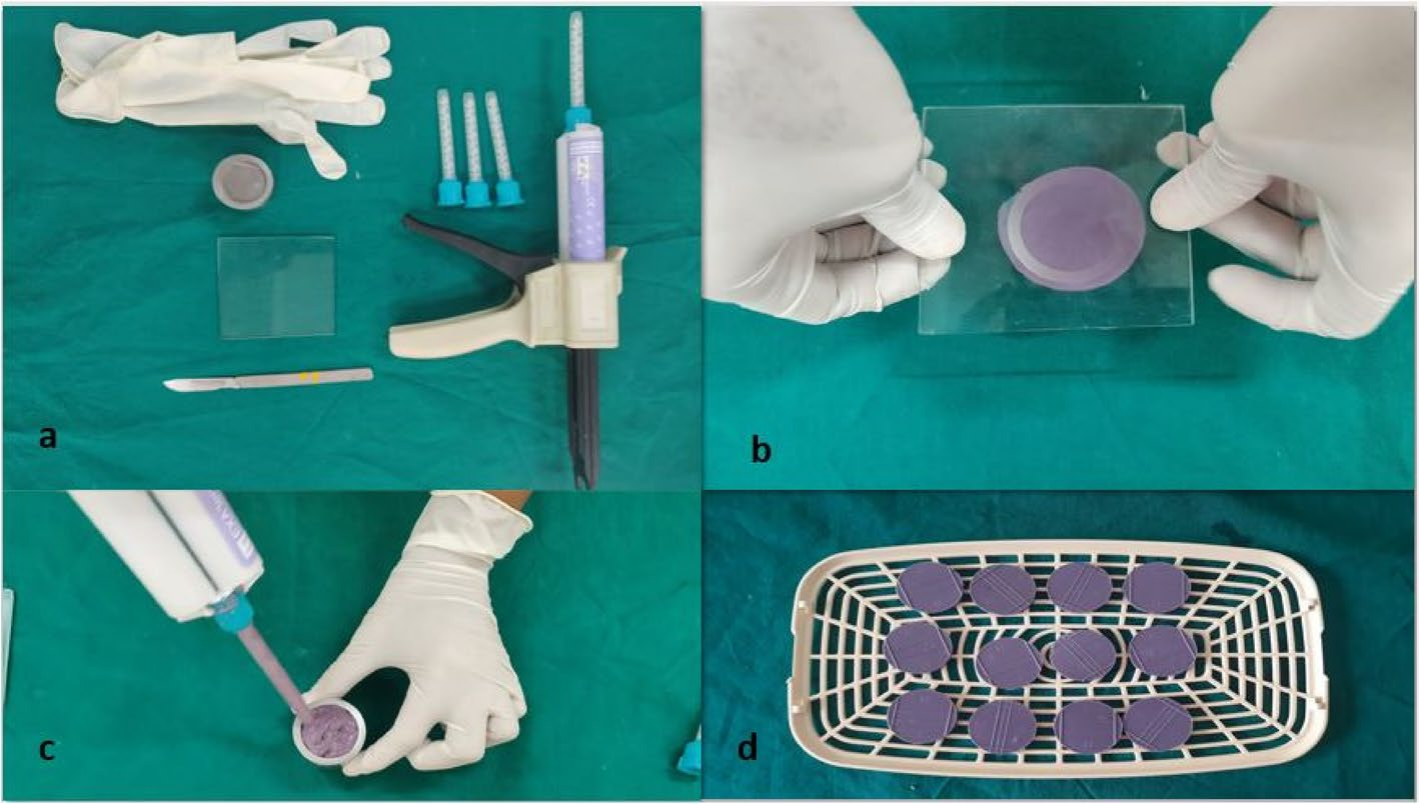
Procedural steps for making samples-**a** Armamentarium for the recording of the impression of metal die; **b** Dispensing of impression material using automixing gun; **c** Glass-plate is placed on top of the metal die; **d** VPES impression samples are kept in a tray for washing under running water

The samples were prepared using EXA'lence Medium Body Monophase Regular Set impression material (Product no. 137444, Lot no. 1607051) by GC America. The metal master die prepared was used for the fabrication of samples. The manipulation of VPES material was done according to the manufacturing instructions. A glass plate (4 × 4 inches square) was positioned on the die and a weight of 1kg was placed over it and impression material was kept for setting for 4–5 mins. The samples were prepared in the form of discs. After the complete set of impression disc, each disc was washed under water for 15 seconds to simulate the clinical working scenario (Fig. 3).

**Fig. 4:**
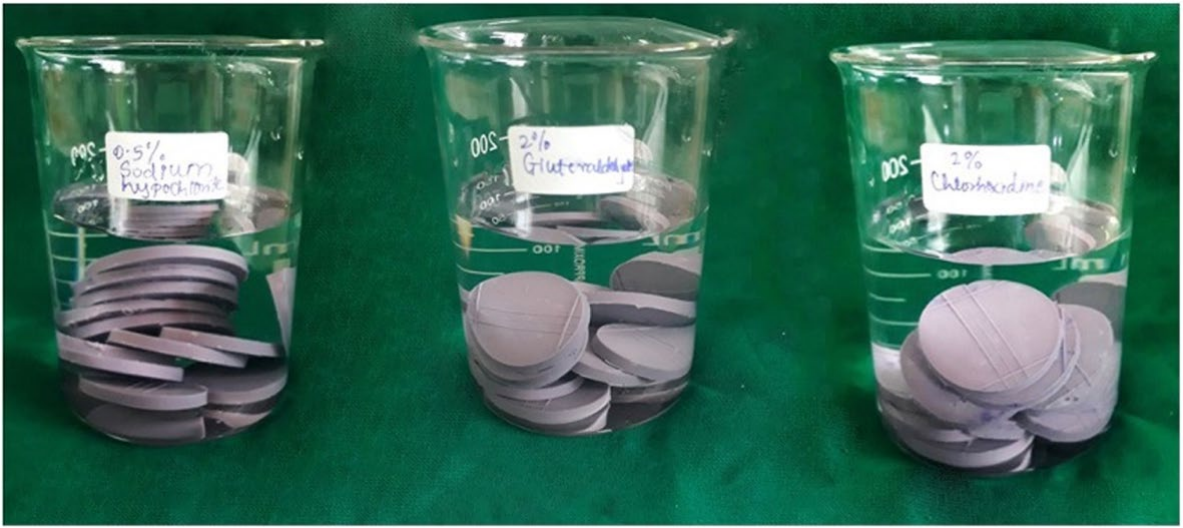
Impression samples immersed in three disinfectant solutions in beakers

In total 84 samples of the impressions in the form of discs were made. In that 24 sample discs were assigned per group, of which 12 discs were randomly allotted for immersion disinfection treatment for a time interval of 10 mins (Fig. 4) and the other 12 for 30 mins in all three groups. 12 samples were allotted for the control group which was not immersed in any disinfectant solution. Their testing was performed under precise predefined laboratory conditions.

These conditions entailed a temperature of 24 ± 2°C. After completing the disinfection protocol, both the test group and control group samples were washed again below the tap water for 10 seconds. This was done to stimulate rinsing of the impression after removal from the disinfectant solution.

### Measurement and recording of the data

**Fig. 5:**
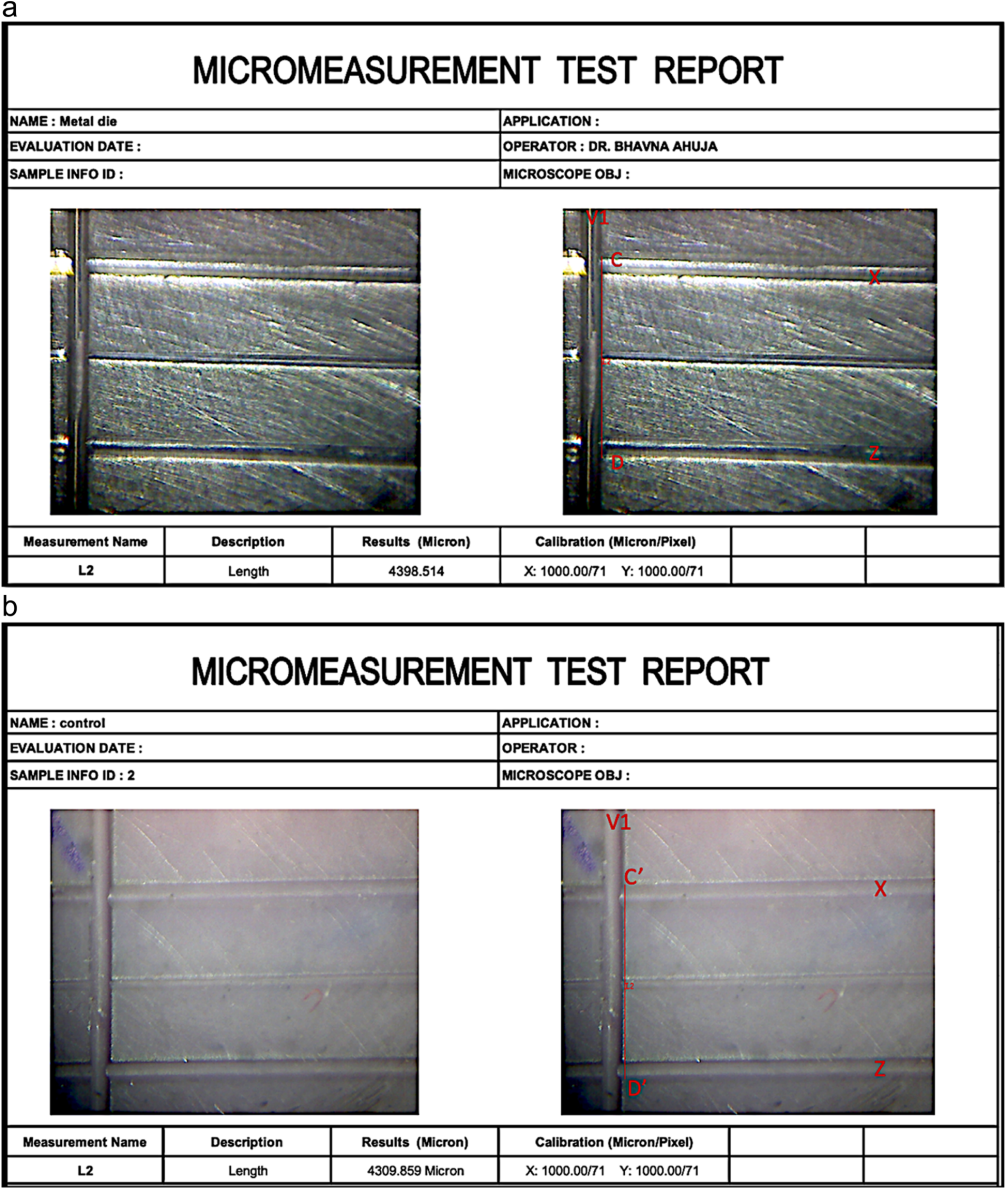
**a** Report showing micro-measurement between the points C-D on Metal die; **b** Report showing micro-measurement between the points C’-D’ on impression disc (Control group)

For the recording of the distance in the sample discs stereomicroscope was used. The distance was measured between the two previously decided points similar to the points on the metal master die- points C and D which were replicated in the samples and were measured at the same points at the intersection of these lines between C’ and D’ by stereomicroscope (Z4 Zoom) (Caliper Pro version - 4.6) with ×40 magnification (0.0001 mm submicron precision). All the measurements were completed by the same researcher both on the Metal die and all the impression discs (Fig. 5a and b). After determining the length, the mean length of all the groups was calculated and later it was further used for comparison of the mean percentage dimensional change using the following equation.

ΔL = 100(L_1_ – L_2_) / L_2_

L_1_ = Mean distance measured between cross lines CD on the metal test block. L_2_ = Distance measured between cross lines on the Impression specimen online C’D’.

### Statistical analysis

The data so obtained was recorded in the Excel sheet and then statistical analysis was performed using SPSS software. Initially, the normal distribution of the data was assessed and then the ANOVA (analysis of variance) test was applied for time intervals of 10 mins and 30 mins followed by Tukey’s post hoc test for multiple comparisons.

## Results

In the present study initial measurement was recorded for the metal master die between X and Z two parallel lines with the stereomicroscope between 2 specific points referred to as C and D which was measured (4398.5 µm). This measurement was taken as a reference and a similar measurement was recorded on all the VPES impression disc samples after disinfection with all three disinfectants for 10 mins and 30 mins.

**Fig. 6:**
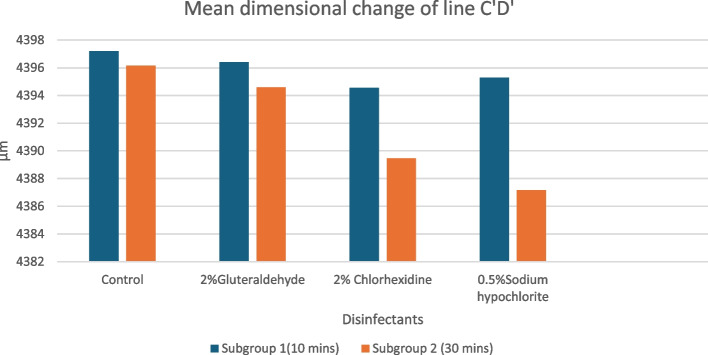
Mean dimensional change of line C’D’

**Fig. 7:**
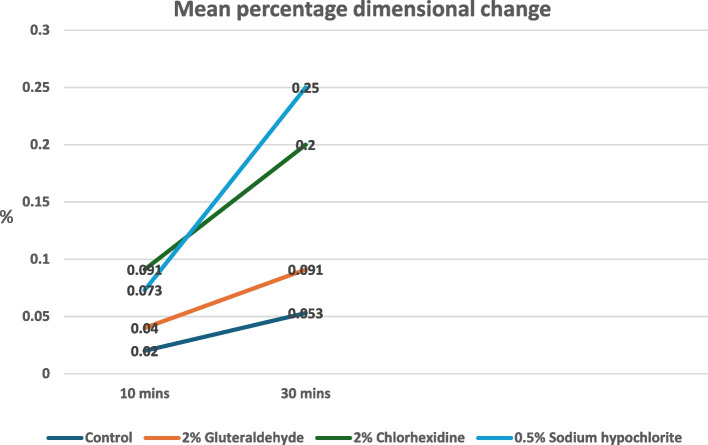
Mean Percentage dimensional change

The distance measured after 10 mins in the control group was 4397.2078 µm; for the test group Group-A- 2% Glutaraldehyde was 4396.4075 µm; Group-B- 0. 5% Sodium hypochlorite was 4394.5453 µm ; Group-C- 2% Chlorhexidine was 4395.2953 µm ; while the distance measured after 30 mins in the control group was 4396.1571 µm; for the test group Group-A- 2% Glutaraldehyde was 4394.5992 µm; Group-B- 0. 5% Sodium hypochlorite was 4389.4711 µm ; Group-C- 2% Chlorhexidine was 4387.1703 µm (Fig. 6) The mean length of all the groups was further used for comparison of the mean percentage dimensional change. The mean percentage dimensional change of the elastomeric material should be near 0.05% [18], this criterion determines the clinical acceptability of the dimensional change of the elastomeric impression material. Percentage dimensional change was in the range of 0.02 – 0.25 % for all the groups for 10 mins and 30 mins (Fig. 7). It is interesting to notice that in Group A (2% Glutaraldehyde), the disinfected and control impression disc exhibited the least increase in contraction from the initial to the 10-minute storage period (0.04%), whereas after 30 mins it showed a significant difference between the two (0.091%). This indicated that immersion of VPES impression material into disinfectants results in the dimensional change of the material, but it is well within the clinically acceptable limits for a period of 10 mins for all the three disinfectants used but after immersion for 30 mins only 2% Glutaraldehyde showed least dimensional change compared to 2% chlorhexidine and 0.5% Sodium hypochlorite, but all were within the clinically acceptable limits. A significant difference was found between the groups in analyzing the data with ANOVA. (for 10 mins *p*=0.00063 ; 30 mins *p*= 0.0001) (Table 1).

**Table 1:** Comparison of dimensional change of elastomeric impression material between three disinfectants after immersion for 10 mins and 30 mins by Analysis of Variance (ANOVA)

	control	2% Glutaraldehyde	2% Chlorhexidine	5% Sodium hypochlorite	Between groups	Sig. *p* value
N	12	12	12	12		
10 mins						
Mean	4397.2078	4396.4075	4394.5453	4395.2953	16.6542	0.00063*
ΣX^2^	232025242	231940801.2	231744370.8	231823471.2		
Std.Dev.	0.9389	1.1398	1.793	1.2928		
30 mins						
Mean	4396.1571	4394.5992	4389.4711	4387.1703	214.6696	0.0001*
ΣX^2^	231914377	231750036.5	231209741.6	230967349.5		
Std.Dev.	1.0473	1.1482	4.9076	4.1246		

∑X- mean square; *p < .05.- significant

## Discussion

Infection control is one of the most important steps to follow in any dental procedure. It is important to maintain the IM disinfection along with dimensional stability [6]. The present study looked at the changes in linear dimensions of VPES (EXA'lence) medium body regular set impression material after disinfection with three different commercially available disinfectants for two different time intervals (10 mins and 30 mins), which are typically used in clinical practice. The results of the study rejected the null hypothesis that there would be no change in dimensional stability in the VPES impressions after immersing it in 3 different disinfectants. It was revealed with the results that the distance between the two points C’D’ measured after disinfection was 4397.2078 µm in the control group, 4396.4075 µm with 2% Glutaraldehyde; 4394.5453 µm with 0. 5% Sodium hypochlorite and 4395.2953 µm with 2% Chlorhexidine for 10 mins of immersion while the distance measured after 30 mins in the control group was 4396.1571 µm; with 2% Glutaraldehyde was 4394.5992 µm; with 0. 5% Sodium hypochlorite was 4389.4711 µm; and with 2% Chlorhexidine was 4387.1703 µm. There was a significant difference in the measurement between the two points among the groups after disinfection (for 10 mins *p*=0.00063 ; 30 mins *p*= 0.0001)*,* which was within the clinically acceptable limits for both time intervals but was least with 2% glutaraldehyde.

AlZain [20] in his systematic review and meta-analysis described various discrepancies between the results of studies conducted to ascertain the impact of disinfection methods on the properties of various impression materials [20]. Disinfecting agents can be used by spraying or immersing methods [12]. The spray method of disinfection has limitations as it does not adequately allow access to difficult-to-reach impression locations like undercut areas and exposure to aerosol to the practitioners. To completely eradicate microbes, the impression should be immersed in disinfection solutions for a predetermined amount of time [15]. The drawback of this method is that it is more time-consuming and every time, a new disinfectant (except for glutaraldehyde) solution is to be made [15, 19].

Recently, a novel substance called VPES was created by combining the two widely used elastomeric impression materials, polyvinyl siloxane (PVS) and polyether (PE). 2009 saw the introduction of this content. The qualities of PVS IM materials include excellent elastic recovery, great dimensional accuracy, tear strength, handling features, and dimensional stability while hydrophobicity is a drawback [1, 18]. PE impression materials, however, yield better impressions since they are naturally hydrophilic. The newly made VPES IM disinfection is also important after clinical use. The most popular method for cleaning dental impressions is chemical disinfection, which includes applying a chemical agent to the impression surface either by immersion or spraying. Glutaraldehyde, sodium hypochlorite (NaOCl), hydrogen peroxide, iodophor, phenol, and chlorine compounds are among the commercially available chemical disinfection products. These products are available in a variety of compositions and quantities. In the present study, 3 regularly used disinfectants among these commercially available materials were used because they have been shown to considerably reduce the number of pathogens on the surface of elastomeric impression materials with little or no change to their physical qualities [21]. The data analysis of previous studies showed that the immersion method was the most often utilised VPES disinfection technique; spray disinfection was only reported in one study in conjunction with the immersion method [22].

The most important cause of dimensional contraction in elastomeric materials is rearrangement and crosslinking of polymer chain links. Other contributing aspects include the loss of water, volatile component loss, and elastic recovery [23].

VPES showed minimal dimensional change after immersion in 2 % Glutaraldehyde for 10 mins as well as 30 mins amongst the experimental groups which were all within the clinically acceptable limit. This was in association with the findings of some of the previous studies [2, 24]. Whereas VPES showed the highest dimensional change after immersion for 10 mins in 2% chlorhexidine interestingly 0.5% sodium hypochlorite showed an increase in mean percentage dimensional change after prolonged storage for 30 mins. Whereas the Control group showed the minimum dimensional change as compared to all experimental groups.

In a study conducted by Nassar U. et al., the impact of glutaraldehyde on VPES impression material was examined. The researchers concluded that a 30-minute immersion in glutaraldehyde had no effect on the material's dimensional stability and that imbibition of the disinfectant's water could have caused the disinfected samples to exhibit less contraction over time, however, continuous polymerization led to an equally increased contraction of all samples over time [24].

Previously few studies evaluated the effect of disinfectants [16, 17] solution concentrations and immersion times on the dimensional accuracy of VPS and PE. In the present study, VPES impression material was evaluated particularly with disinfectants such as 0.5% sodium hypochlorite and 2% chlorhexidine. The results revealed that the dimensional change was within the clinically acceptable limits for both the immersion times (10 mins and 30 mins) assessed. It was revealed from this study that immersion of VPES in these disinfectants leads to more mean contraction of impression discs as compared to that of 2% Glutaraldehyde. The change in the dimension was minimum for 2% Glutaraldehyde, which suggests that this disinfectant is most suitable for VPES impression material. The impressions made from VPES if disinfected with the 2% Glutaraldehyde would produce the cast with maximum accuracy.

Glutaraldehyde and sodium hypochlorite are frequently used for immersion disinfection techniques. Compared to glutaraldehyde, NaOCl produced more dimensional variations in PVES. In the systematic review on VPES IM, it was documented that NaOCl-mediated disinfection was linked to significant dimensional variability in VPES impressions, despite the overall results for immersion disinfection showing no significant effect on dimensional variations. When calculating disinfectant-induced dimensional changes, the immersion time is a key factor, storage time after which the measurements were taken [22]. The type of solution used for the immersion of dental prosthesis material determines the degree of dimensional changes in dental impressions; nevertheless, evidence from published studies about other elastomeric impression materials shows that immersion time has no clinically relevant effect on these changes [22]. These findings were in correlation with the results of the present study as the dimensional changes in the test samples were within clinically acceptable range both for 10 mins and 30 mins. A thirty-minute glutaraldehyde disinfection time was recorded in only one investigation, whereas a three-minute Cavex disinfection time was observed in another [22, 25]. The immersion technique with a ten-minute disinfection interval was employed in most of the studies. Chemical disinfection has little effect on the dimensional stability of VPES, as described in the findings of a meta-analysis of a review [22]. The current study's findings demonstrated that all groups, including the control group, had clinically acceptable dimensional changes even after 30 minutes with the least change in dimension in 2% glutaraldehyde.

Compared to polyether, silicon-based IM materials often withstand immersion disinfection better in terms of dimensional stability. The dimensional integrity of VPES dental impressions may be harmed by various immersion solutions rather than disinfectants because PVES is a combination of silicones and polyether.

According to previous studies, several disinfectants and PVS immersion times have demonstrated good compatibility and are dimensionally stable for up to 18 hours of immersion time [26]. When PE (medium body) was disinfected by either a 10-minute immersion or a one-hour immersion in a 0.5% sodium hypochlorite solution, all disinfection times showed expansion [27].

The clinical implication of this study can be stated that 2 % glutaraldehyde gives satisfactory disinfection of VPES elastomeric impression material and immersion for normal patients for the time interval of 10 mins is sufficient to be followed and for immunocompromised patients, 30 mins can be used with the minimum dimensional change of VPES elastomeric impression material.

The limitations of this study are that only the immersion method of disinfection is used in the study other disinfection methods like using spray atomization or ultraviolet disinfection or autoclaving or microwaving of VPES impression material should also be checked for its effect on the dimensional stability of the impression material. Future studies are recommended with the assessment of microbe growth in the VPES impression materials, more clinical studies would be beneficial for better and clinical-oriented results.

## Conclusions

Within the limitations of the study, it can be concluded that VPES impression material can be effectively disinfected within clinically acceptable limits with 2% glutaraldehyde. 2 % Glutaraldehyde is the most suitable disinfectant for immersion periods of 10 mins and 30 mins for VPES elastomeric impression material and shows minimum dimensional changes as compared when immersed in 2% Chlorhexidine and 0.5% Sodium hypochlorite.

## Data Availability

The datasets used and/or analysed during the current study are available from the Primary author upon reasonable request.

## Abbreviations

VPES: Vinyl polyether silicone
PVES: Polyvinyl ether siloxane
NaOCl: Sodium hypochlorite
ADA: American Dental Association
PVS: Polyvinyl siloxane
PE: Polyether
ANOVA: Analysis of variance
SS: Stainless steel
MMD: metal master die
